# Phylogenetic relationships among *Bradyrhizobium* species nodulating groundnut (*Arachis hypogea* L.), jack bean (*Canavalia ensiformis* L.) and soybean (*Glycine max* Merr.) in Eswatini

**DOI:** 10.1038/s41598-022-14455-9

**Published:** 2022-06-23

**Authors:** Zanele D. Ngwenya, Mustapha Mohammed, Sanjay K. Jaiswal, Felix D. Dakora

**Affiliations:** 1 Department of Crop Sciences, Tshwane University of Technology, Private Bag X680, Pretoria, 001 South Africa; 2 Present Address: Department of Chemistry, Tshwane University of Technology, Private Bag X680, Pretoria, 001 South Africa; 3 Department of Crop Science, University for Development Studies, P.O. Box 1350, Tamale, Ghana

**Keywords:** Microbiology, Bacteria, Symbiosis

## Abstract

This study assessed the genetic diversity and phylogenetic relationships of rhizobial isolates obtained from root nodules of groundnut, jack bean and soybean planted in different locations within Eswatini. Seventy-six rhizobial isolates were studied using ERIC-PCR (enterobacterial repetitive intergenic consensus) fingerprinting and PCR amplification of 16S rRNA, housekeeping genes (*atpD, dnaK, glnll* and *rpoB*) and symbiotic genes (*nifH* and *nodC*). The dendrogram generated from the ERIC-PCR banding patterns grouped the test rhizobial isolates into 16 major clusters (Cluster I–XVI), with three isolates, namely TUTAHeS60, TUTGMeS3 and TUTAHeS127, forming outgroups of Clusters IV, VI and IX, respectively. Furthermore, the 76 test isolates were grouped into 56 ERIC-PCR types at 70% similarity level. The phylogenetic analysis of the 16S rRNA gene and multilocus sequence analysis of four housekeeping (*atpD, dnaK, glnII* and *rpoB*) and two symbiotic (*nifH* and *nodC*) genes showed that all three legumes (groundnut, jack bean and soybean) were nodulated by bacterial symbionts belonging to the genus *Bradyrhizobium*, with some isolates exhibiting high divergence from the known reference type strains. The results also showed that *B. arachidis, B. iriomotense* and *B. canariense* were the closest type strains to the groundnut isolates, while *B. pachyrhizi* and *B. elkanii* were the closest relatives to the bacterial symbionts associated with the nodulation of both jack bean and soybean. This study is the first report to describe of the bacterial symbionts nodulating jack bean in African soils.

## Introduction

In legumes, Biological Nitrogen Fixation (BNF) occurs via a symbiotic relationship with soil bacteria collectively known as “rhizobia”^1^. Rhizobial symbionts of legumes are phylogenetically diverse and are distributed in fifteen bacterial genera including *Bradyrhizobium, Azorhizobium*, *Ensifer* (*Sinorhizobium*), *Mesorhizobium, Rhizobium*, *Burkholderia* and *Cupriavidus* among others^2^. Although these diverse rhizobia may share similar morphological and cultural characteristics, they sometimes exhibit some level of host specificity^3^. For example, groundnut (*Arachis hypogea*) is generally nodulated by both slow and fast-growing rhizobia belonging to the *Rhizobium* and *Bradyrhizobium* genera^4–7^. On the other hand, soybean is predominantly nodulated by rhizobial species in the *Bradyrhizobium* genus, and to a lesser extent by species belonging to the genera *Rhizobium, Mesorhizobium* and *Sinorhizobium*^8–10^.

Exploring the types of rhizobia nodulating different legumes is one of the steps towards harnessing the benefits of their N_2_-fixing trait for improved crop production through their formulation into inoculants^11^. Earlier studies have reported the phylogenetic relationships among symbionts of cultivated legumes such as soybean^4,12,13^ and groundnut^5^ in Africa. These reports have shown that the dominant symbionts of these legumes are genetically diverse species in the *Bradyrhizobium* genus. Several authours have carried out bacterial phylogenetic analyses through analyses of the 16S ribosomal RNA (16S rRNA) gene sequences, a range of “housekeeping” and symbiotic genes^2,14^. While it is important to continue exploring the symbionts of cultivated legumes in different environments, it is equally crucial to study rhizobia nodulating underutilized legumes such as the jack bean (*Canavalia ensiformis*) in the hope of discovering super rhizobia. Despite the high yield potential and food value of jack bean, this legume is neglected by researchers and producers. As a result, little information exists on the microsymbionts nodulating jack bean.

The aim of this study was to assess the genetic diversity and the phylogenetic relationships among rhizobial symbionts of two important cultivated grain legumes in Africa (groundnut and soybean), and an underutilized legume (jack bean) at different locations in Eswatini, using ERIC-PCR fingerprinting, multilocus sequence analysis of the 16S rRNA gene, housekeeping (*atpD, dnaK, glnll* and *rpoB*) and symbiotic genes (*nifH* and *nodC*).

## Materials and methods

### Source of root nodules

The groundnut nodules were collected from field-grown plants at the Malkerns Research Station in Eswatini with map coordinates 26° 33′ S, 31° 10′ E, while jack bean and soybean nodules were obtained through trapping in the glasshouse using soils from Ka-Zulu and New Heaven with map coordinates 26° 45′ S, 31° 15′ E and 27°03′ S, 31°29′ E, respectively. To trap the rhizobia, soils from four different locations in Eswatini were used to inoculate seedlings of jack bean and soybean grown aseptically in sterile sand in the glasshouse. The plants were watered with sterile N-free nutrient solution until harvesting at 60 days after planting (DAP). The chemical properties of the soils from the various locations are presented in Supplementary Table S1.

### Trapping rhizobia from field soils in the glasshouse

Where nodules could not be obtained from field grown plants, soils were sampled from Malkerns Research Station, Ka-Zulu, Luve and New Heaven in Eswatini for trapping rhizobia under glasshouse conditions. Jack bean accession 493 and five soybean genotypes (TGx200-25DM, TGx1830-20E, TGx2001-25DM, TGx1988-9F and PAN 1614) were used as host plant to trap rhizobia. Three surface sterilised seeds of each legume were planted per pot, with three replications per location. After germination, seedlings were thinned to one seedling per pot, with three replicate pots per soil type. Soil inocula were prepared by adding 20 g of each soil sample to 100 mL sterile distilled water and the soil suspension used to inoculate the seedlings. To avoid the possible suppression of nodulation by nitrogen fertilization, the plants were irrigated with N-free nutrient solution^15^ in alternation with sterile water. Thus, the plants relied solely on symbiosis to meet their N requirements. The plants were harvested at 60 DAP and root nodules plucked and stored in silica gel at 4 °C prior to bacterial isolation within one week. We can confirm that the use of plants in different aspects of this study complied with international, national and/or institutional guidelines.

### Isolation of bacteria from root nodules

The nodules were rehydrated in water for two hours, rinsed with sterile distilled water and surface-sterilised by immersing in 95% ethanol for 5 s followed by transferring to a 3% sodium hypochlorite solution for 5 min. The nodule was then rinsed 5–6 times with sterile distilled water. Each surface-sterilised nodule was crushed in a sterile petri dish, and the nodule suspension was streaked on yeast-mannitol agar (YMA) plate^16^. The plates were sealed using parafilm and incubated at 28 °C for 7–10 days. Bacterial growth on the YMA plates was observed daily for the appearance of single colonies. Colony characteristics such as colour, texture, size (diameter) and appearance were recorded. For long-term storage, cultures of pure single colonies were stored in 30% glycerol at − 20 °C^17^.

### Authentication of bacterial isolates

The bacterial isolates were used to inoculate their respective homologous hosts in the glasshouse and observed for nodulation in fulfilment of Koch’s postulate. The plants were watered with sterile N-free nutrient solution^15^, and alternated with sterile water where necessary. Three replicate pots were used per isolate. Uninoculated plants were included as control. The plants were harvested at 60 days after planting and assessed for nodulation.

### Bacterial genomic DNA extraction and ERIC PCR amplification

To extract bacterial genomic DNA, the isolates were cultured in YM broth to late log phase. DNA was extracted using the GenElute bacterial genomic DNA extraction kit (Sigma-Aldrich, USA) according to the manufacturer’s instructions. The extracted DNA were subjected to ERIC (enterobacterial repetitive intergenic consensus) PCR using universal primers (Supplementary Table S2). A 25 µL PCR mix volume was prepared containing 12.5 µL 2 × myTaq PCR master mix, 1 μL each of the primer pair (Supplementary Table S2), 9.5 μL nuclease-free PCR water and 1 μL (40–50 ng μL^−1^) extracted DNA. The PCR amplification was performed in a Thermal cycler (T100 BIORAD, USA).

**Table 1:** Host genotype and morphological characteristics (colour, shape, texture, size, growth, and appearance) of groundnut, jack bean and soybean isolates used in this study.

Isolates	Cluster (≥ 70% similarity)	Major cluster	Host genotype	Colour	Shape	Texture	Size (mm)	Growth days	Appearance
TUTCEeS10	1	I	CV. Accession 502^**JB**^	Milky	Flat	Gummy	1	7	Opaque
TUTCEeS11	1		CV. Accession 503^**JB**^	Milky	Flat	Gummy	1	4	Opaque
TUTCEeS17	1		CV. Accession 509^**JB**^	Milky	Flat	Gummy	1	1	Opaque
TUTGMeS28	2		TGx1830-20E^**SB**^	Milky	Flat	Gummy	1	10	Opaque
TUTAHeS90	3		CV. Natal common^**GD**^	White	Doomed	Gummy	2	26	Translucent
TUTAHeS91	3		CV. Natal common^**GD**^	White	Doomed	Gummy	6	18	Translucent
TUTAHeS95	3		CV. Natal common^**GD**^	White	Doomed	Gummy	4	9	Translucent
TUTAHeS17	4	II	CV. Natal common^**GD**^	White	Flat	Gummy	1	7	Translucent
TUTCEeS38	4		CV. Accession 514^**JB**^	White	Flat	Watery	1	7	Translucent
TUTAHeS55	5		CV. Natal common^**GD**^	White	Doomed	Gummy	4	9	Translucent
TUTCEeS4	6	III	CV. Accession 496^**JB**^	Milky	Doomed	Gummy	6	11	Opaque
TUTGMeS21	7		TGx1830-20E^**SB**^	White	Flat	Watery	2	10	Translucent
TUTAHeS29	8		CV. Natal common^**GD**^	White	Flat	Watery	1	6	Translucent
TUTAHeS65	8		CV. Natal common^**GD**^	White	Flat	Watery	1	2	Translucent
TUTAHeS27	9		CV. Natal common^**GD**^	Milky	Flat	Gummy	2	15	Opaque
TUTGMeS17	10	IV	TGx1830-20E^**SB**^	White	Flat	Watery	2	8	Translucent
TUTGMeS30	10		TGx1830-20E^**SB**^	White	Flat	Watery	1	8	Translucent
TUTGMeS19	11		TGx1830-20E^**SB**^	Milky	Flat	Watery	4	8	Opaque
TUTAHeS72	12		CV. Natal common^**GD**^	White	Flat	Watery	2	7	Translucent
TUTAHeS82	13		CV. Natal common^**GD**^	Milky	Doomed	Gummy	2	3	Opaque
TUTAHeS60	14	Outgroup of IV	CV. Natal common^**GD**^	Milky	Flat	Gummy	2	7	Opaque
TUTGMeS24	15	V	TGx1830-20E^**SB**^	White	Flat	Watery	1	8	Translucent
TUTGMeS26	15		TGx1830-20E^**SB**^	White	Flat	Watery	1	6	Translucent
TUTGMeS16	16		TGx2001-25DM^**SB**^	White	Flat	Watery	1	14	Translucent
TUTGMeS18	16		TGx1830-20E^**SB**^	White	Flat	Watery	1	3	Translucent
TUTGMeS1	17		TGx1988-9F^**SB**^	White	Flat	Watery	1	14	Translucent
TUTGMeS2	17		TGx1830-20E^**SB**^	White	Flat	Watery	1	7	Translucent
TUTGMeS10	18	VI	TGx2001-25DM^**SB**^	White	Flat	Watery	2	10	Translucent
TUTGMeS22	18		TGx1830-20E^**SB**^	White	Flat	Watery	2	6	Translucent
TUTGMeS3	19	Outgroup of VI	TGx1830-20E^**SB**^	White	Flat	Watery	2	10	Translucent
TUTGMeS7	20	VII	TGx2001-25DM^**SB**^	White	Flat	Watery	1	8	Translucent
TUTGMeS9	20		TGx2001-25DM^**SB**^	White	Flat	Watery	1	10	Translucent
TUTGMeS29	20		TGx1830-20E^**SB**^	White	Flat	Watery	2	10	Translucent
TUTGMeS31	21		TGx1830-20E^**SB**^	Milky	Flat	Watery	1	7	Opaque
TUTCEeS3	22	VIII	CV. Accession 495^**JB**^	White	Flat	Watery	1	10	Translucent
TUTCEeS6	22		CV. Accession 498^**JB**^	White	Flat	Watery	1	4	Translucent
TUTCEeS7	22		CV. Accession 499^**JB**^	White	Flat	Watery	1	11	Translucent
TUTCEeS13	22		CV. Accession 505^**JB**^	White	Flat	Watery	1	14	Translucent
TUTCEeS14	22		CV. Accession 506^**JB**^	White	Flat	Watery	1	14	Translucent
TUTCEeS9	23		CV. Accession 501^**JB**^	White	Flat	Watery	1	14	Translucent
TUTCEeS5	24		CV. Accession 497^**JB**^	White	Flat	Watery	1	6	Translucent
TUTAHeS130	25	IX	CV. Natal common^**GD**^	Milky	Flat	Watery	2	7	Opaque
TUTAHeS167	26		CV. Natal common^**GD**^	Milky	Flat	Watery	1	8	Opaque
TUTCEeS15	27		CV. Accession 507^**GD**^	White	Flat	Watery	1	14	Translucent
TUTCEeS16	27		CV. Accession 508^**GD**^	White	Flat	Watery	1	13	Translucent
TUTCEeS1	28		CV. Accession 493^**GD**^	White	Flat	Watery	1	12	Translucent
TUTCEeS2	28		CV. Accession 494^**GD**^	White	Flat	Watery	2	12	Translucent
TUTAHeS127	29	Outgroup of IX	CV. Natal common^**GD**^	Milky	Doomed	Gummy	2	4	Opaque
TUTCEeS18	30	X	CV. Accession 510^**JB**^	Milky	Flat	Gummy	1	4	Opaque
TUTCEeS8	31		CV. Accession 500^**GD**^	White	Flat	Watery	1	12	Translucent
TUTAHeS26	32		CV. Natal common^**GD**^	White	Flat	Gummy	1	15	Translucent
TUTCEeS36	33	XI	CV. Accession 513^**GD**^	Milky	Flat	Gummy	1	4	Opaque
TUTAHeS131	34		CV. Natal common^**GD**^	Milky	Flat	Watery	1	8	Opaque
TUTGMeS32	35	XII	TGx1830-20E^**SB**^	White	Flat	Watery	1	7	Translucent
TUTAHeS67	36		CV. Natal common^**GD**^	Milky	Flat	Watery	1	12	Opaque
TUTCEeS12	37		CV. Accession 504^**JB**^	White	Doomed	Watery	6	13	Translucent
TUTAHeS70	38		CV. Natal common^**GD**^	White	Doomed	Gummy	2	7	Translucent
TUTGMeS8	39	XIII	TGx2001-25DM^**SB**^	Milky	Flat	Watery	1	10	Opaque
TUTAHeS125	40		CV. Natal common^**GD**^	Milky	Flat	Watery	1	7	Opaque
TUTAHeS4	41		CV. Natal common^**GD**^	Milky	Doomed	Gummy	2	12	Opaque
TUTGMeS25	42	XIV	TGx1830-20E^**SB**^	White	Flat	Watery	1	8	Translucent
TUTGMeS27	42		TGx1830-20E^**SB**^	White	Flat	Watery	2	3	Translucent
TUTGMeS13	43		TGx2001-25DM^**SB**^	Milky	Flat	Watery	1	10	Opaque
TUTGMeS23	44		TGx1830-20E^**SB**^	White	Flat	Watery	1	8	Translucent
TUTAHeS3	45		CV. Natal common^**GD**^	Milky	Flat	Gummy	1	5	Opaque
TUTGMeS14	46		TGx2001-25DM^**SB**^	White	Flat	Watery	1	8	Translucent
TUTCEeS21	47	XV	CV. Accession 512^**JB**^	Milky	Flat	Watery	1	6	Opaque
TUTAHeS123	48		CV. Natal common^**GD**^	Milky	Doomed	Gummy	3	7	Opaque
TUTCEeS19	49		CV. Accession 511^**JB**^	Milky	Flat	Watery	1	7	Opaque
TUTGMeS33	50		TGx1830-20E^**SB**^	Milky	Flat	Watery	1	7	Opaque
TUTGMeS5	51		TGx1830-20E^**SB**^	Milky	Flat	Gummy	1	7	Opaque
TUTGMeS20	52		TGx1830-20E^**SB**^	White	Flat	Watery	2	10	Translucent
TUTAHeS13	53	XVI	CV. Natal common^**GD**^	Milky	Flat	Watery	2	8	Opaque
TUTAHeS23	54		CV. Natal common^**GD**^	Milky	Doomed	Gummy	4	11	Opaque
TUTGMeS4	55		TGx1830-20E^**SB**^	Milky	Doomed	Gummy	1	10	Opaque
TUTGMeS6	56		TGx1830-20E^**SB**^	Milky	Doomed	Gummy	2	10	Opaque

Growth refers to the number of days taken for colonies to appear on yeast mannitol agar plates while size represents colony dimeter.

Species of host genotype are defined by superscripts as; groundnut (*Arachis hypogea*) = ^**GD**^; jack bean (*Canavalia ensiformis*) = ^**JB**^ and soybean (*Glycine max*) = ^**SB**^.

The amplified products were subjected to gel electrophoresis in 1.2% agarose gel stained with ethidium bromide. The PCR was run at 85 V for 6 h. The gel images were recorded using the GEL DocTM 186 XR + molecular imager (Bio-RAD, USA). Cluster analysis was done to generate a dendrogram using the unweighted pair group method with arithmetic mean (UPGMA) algorithm using the software Bionumerics (version 8).

### Amplification of the 16S rRNA, housekeeping (*atpD, dnaK, glnll, rpoB*) and symbiotic (*nifH, nodC*) genes

The PCR amplification of the 16S rRNA, housekeeping genes (*atpD, dnaK, glnll and rpoB*) and symbiotic genes (*nifH* and *nodC*) were individually performed in a 25 µL PCR reaction volume, which contained 1 µL DNA (50 to 70 ng µL^−1^), 3 µL MyTaq buffer (5×), 1 µL each of forward and reverse primers of the gene of interest (Supplementary Table S2), 0.1 µL Taq polymerase (5U) (Bioline, USA), and 18.9 μL double distilled ultrapure water. The PCR was performed in a Bio-Rad T100 thermal cycler using standard temperature profiles (Supplementary Table S2). The amplified products were confirmed by gel electrophoresis in a 1.2% agarose gel stained with ethidium bromide in TAE buffer at 85 V for 2 h. The gel images were photographed using UV illumination with a gel documentation system (BIO-RAD Gel DocTM XR+).

### Sequence and phylogenetic analyses

Prior to sequencing, the amplified PCR products were purified using a PCR Cleanup kit (NEB, USA), according to the manufacturer’s guidelines. Thereafter, the amplified PCR products were sequenced at Macrogen (Netherlands). The quality of the sequences was assessed using the software BioEdit 7.0.9.0^18^. Closely related species were identified using the BLASTn program in the NCBI (National Centre for Biotechnology Information) database. Multiple and pairwise sequence alignments were carried-out using CLUSTALW, and phylogenetic trees were constructed using MEGA 7 software by means of the maximum likelihood statistical method^19^. The robustness of the tree branching was estimated using 1000 bootstrap replicates^20^. The sequences obtained were deposited in the NCBI GenBank to obtain accession numbers OM721967–OM721998 (*16S rRNA*), OM744177–OM744199 (*atpD*), OM839789–OM839804 (*dnaK*), OM839805–OM839832 (*glnII*), OM839833–OM839864 (*rpoB*), OM839865–OM839870; OM839872-OM839876; OM839882-OM839884 (*nifH*) and OM846520–OM846539 (*nodC*).

### Statistical analysis

The pH and other chemical properties of soils from the various test locations were transformed into a matrix using principal component analysis (PCA). Out of 10 PC axes, the first two were used for clear visualization of the data. Data were analyzed in the R platform using the libraries ‘‘FactoMineR”, ‘‘factoextra” and ‘‘corrplot”^21–24^.

## Results

### ERIC-PCR fingerprints of groundnut, jack bean and soybean rhizobial isolates

A total of 133 bacterial isolates were obtained from the root nodules of groundnut, of which 24 could form root nodules on the homologous host. Of the 34 bacterial isolates from the root nodules of jack bean, 22 nodulated the host plant in an authentication study, while 30 out of 48 bacterial isolates from soybean induced root nodules on the host plant. Thus, there were a total of 76 authenticated rhizobial isolates from the three test legume species. These rhizobial isolates showed differences in growth rate, colony colour, shape, texture, size and appearance (Table 1). The non-rhizobial endophytes isolated were stored for further studies.

**Figure 1:**
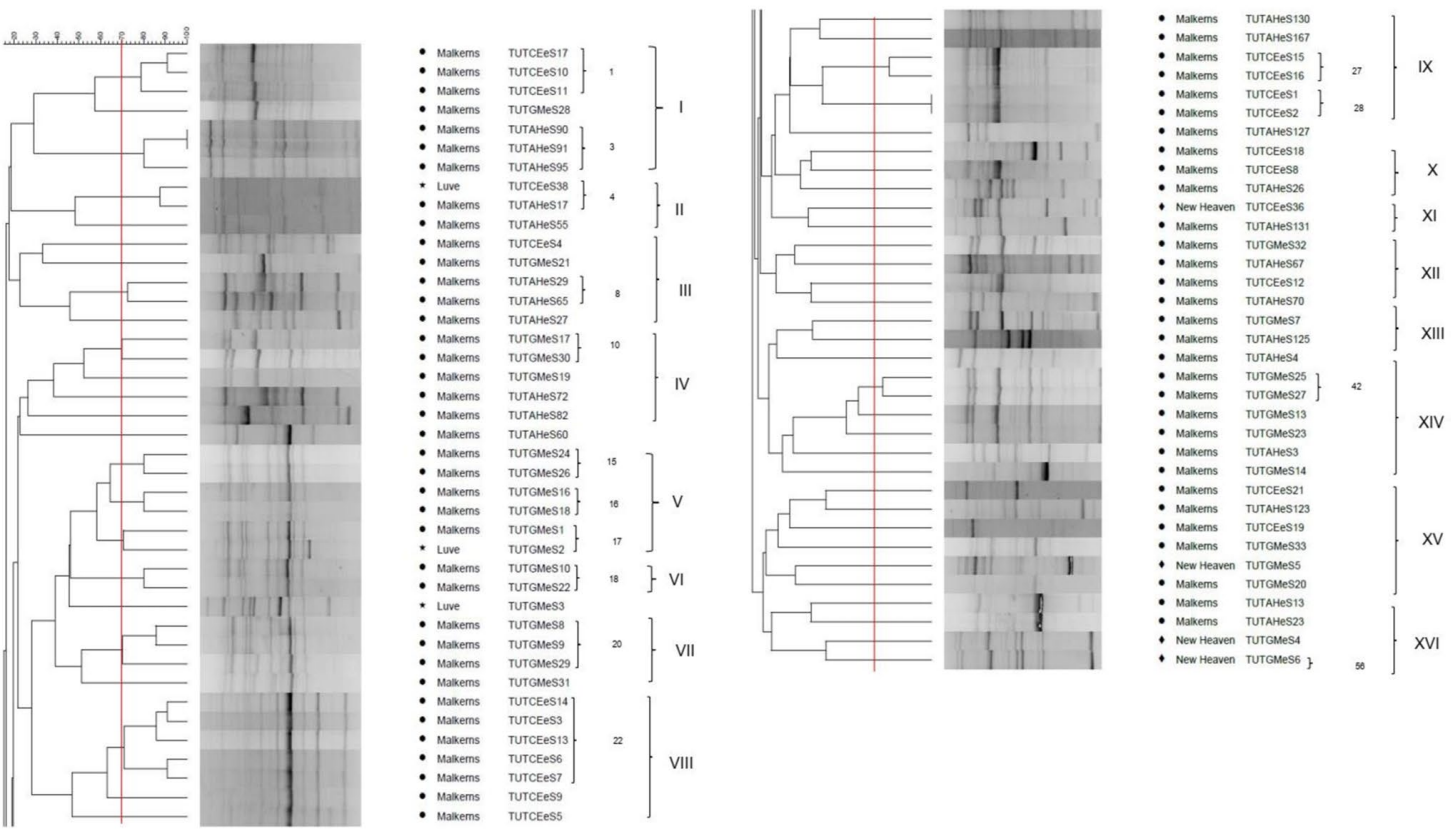
Dendrogram of ERIC-PCR fingerprints obtained from 76 rhizobial isolates from groundnut, jack bean and soybean from various locations in Eswatini.

Subjecting the ERIC-PCR products of the 76 rhizobial isolates to gel electrophoresis yielded different band sizes, which ranged from 500 to 8000 bp. The dendrogram generated from the ERIC-PCR banding patterns grouped the test rhizobial isolates into 16 major clusters (I–XVI), with three isolates (namely, TUTGMeS3, TUTAHeS60 and TUTAHeS127) forming outgroups of Clusters II, IV and IX, respectively. The 76 test isolates grouped into 56 ERIC-PCR types if considered at a 70% similarity cut-off point (Fig. 1).

Isolates from Cluster I, III, XII and XV were more heterogeneous in composition, and comprised microsymbionts from soybean, jack bean and groundnut (Fig. 1; Table 1). Cluster II on the other hand contained two groundnut isolates from the Malkerns Research Station and one jack bean isolate from Luve (Fig. 1; Table 1). Cluster IV consisted of three jack bean isolates and two groundnut isolates from the Malkerns Research Station (Fig. 1; Table 1). Isolates in Cluster VI and VII contained two and four soybean isolates, respectively, all from the Malkerns Research Station, while Cluster V contained five soybean isolates from the Malkerns Research Station and one soybean isolate from Luve. Seven jack bean isolates from Malkerns also grouped together to form cluster VIII (Fig. 1; Table 1). Cluster IX, X and XI contained isolates from both jack bean and groundnut, while Clusters XIII, XIV and XVI contained rhizobial isolates from the root nodules of soybean and groundnut (Fig. 1; Table 1). Clusters II, V, XI, XV and XVI consisted of isolates from different locations while Clusters I, III, IV, VI, VII, VIII, IX, X, XII, XIII and XIV contained isolates from a single location (i.e., Malkerns Research Station). Of the 30 soybean isolates in the dendrogram, 19 were from the root nodules of genotype TGx1830-20E, while seven were from the soybean genotype TGx1988-9F, and four from TGx2001-25D (Table 1).

### Influence of soil chemical properties on the distribution of rhizobia

**Figure 2:**
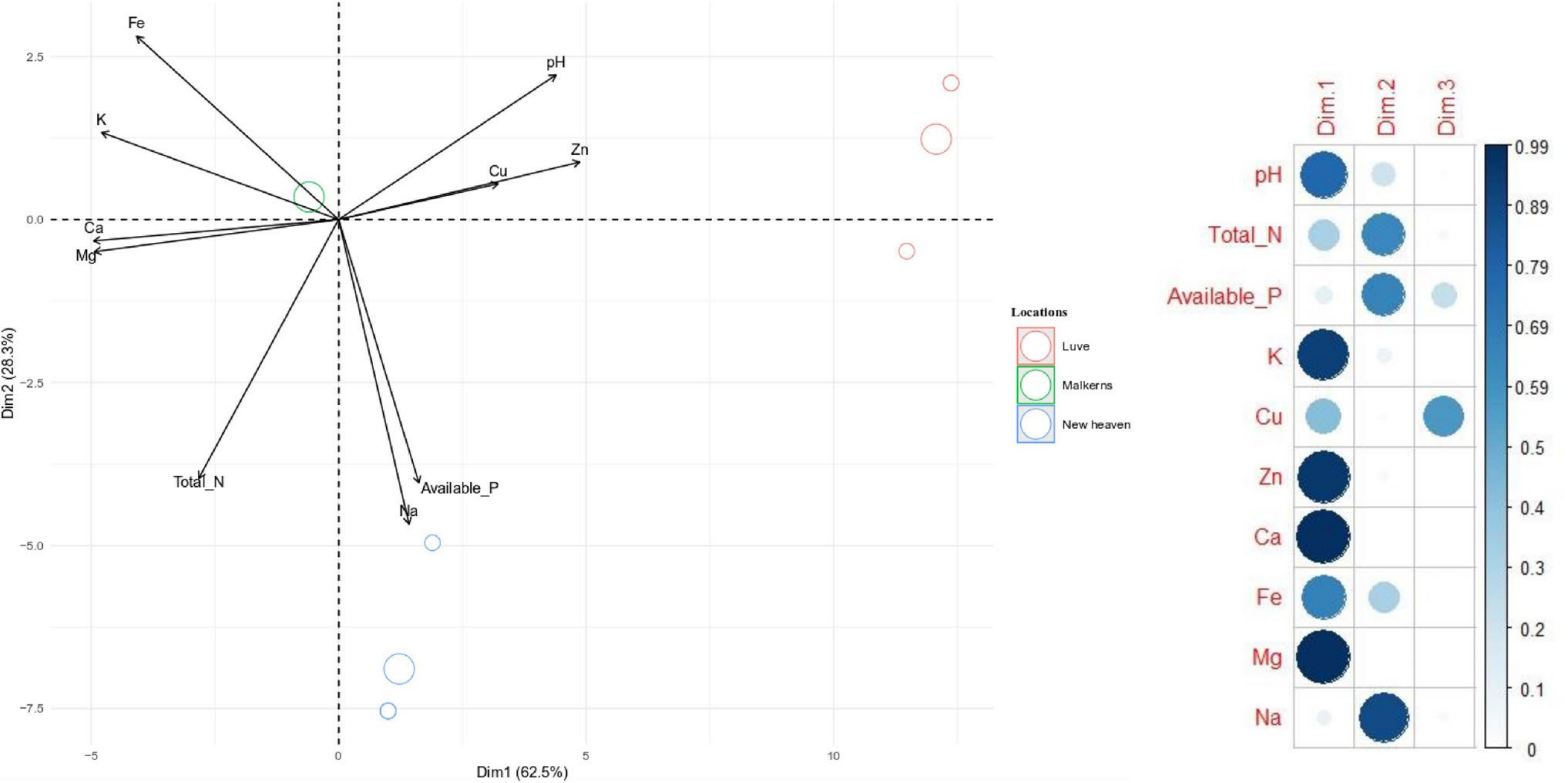
A PCA analysis showing the influence of soil chemical parameters on the distribution of rhizobial symbionts of groundnut, jack bean and soybean from three locations (Malkerns, Luve and New Heaven) in Eswatini.

The Principal Component Analysis (PCA) was done to assess the influence of soil chemical properties on the distribution of microsymbionts across the test locations (Fig. 2). The results revealed that dimension 1 (PC1) and dimension 2 (PC2) accounted for more than 90% of the explained variables. PC1 explained most of the soil chemical properties (Fig. 2). The PCA results showed that the soil variables correlated with underlying microsymbiont diversity between locations. The pH, Total N, K, Cu, Zn, Ca, Fe and Mg of soils from the various agroecological zones of ESwatini were highly linked and positively correlated to PC1 (Fig. 2). The levels of Ca, K, Mg and Fe in soils highly influenced the distribution of microsymbionts collected from Malkerns, and contained a high value for the second principal component, while soil available P and Na showed negative with PC2 and the microsymbionts obtained from New heaven. The results further showed that the isolates from Luve were more influenced by soil pH, Zn and Cu (Fig. 2).

### Phylogenetic relationships of isolates inferred from 16S rRNA gene sequences

**Figure 3:**
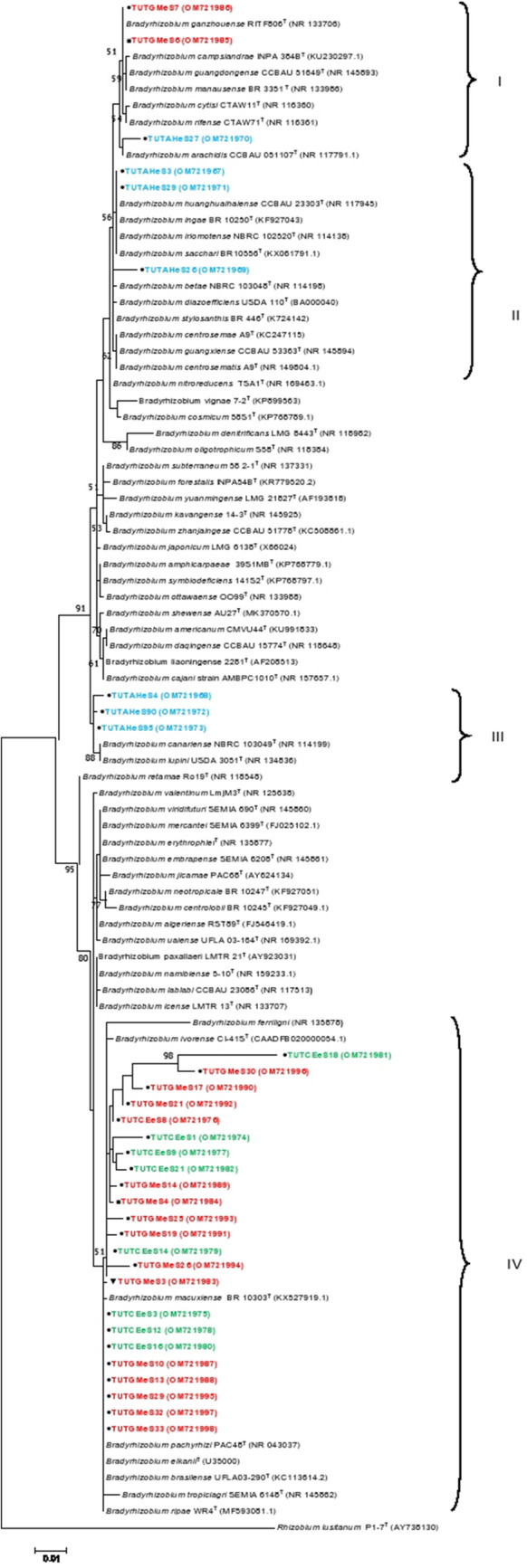
Maximum likelihood phylogeny of rhizobial symbionts of groundnut, jack bean and soybean from various locations in Eswatini based on 16S rRNA gene sequences. For each isolate, the location of origin is indicated by assigning different symbols, e.g., circle-Malkerns Research station, square-New Heaven and triangle-Luve. GenBank accession numbers are indicated in parenthesis after the name of each isolate. Isolates’ names are colour coded based on the host species as Blue-groundnut, Green-jack bean and Red-soybean.

For phylogenetic analysis, 32 representative isolates from the three legume species were randomly selected from the different ERIC-PCR clusters for sequence analysis of the 16S rRNA gene. The PCR amplified products of the gene were approximately 1200 bp in length. However, a final length of 965 bp was used for phylogenetic analysis after alignment with reference strains and trimming. The maximum-likelihood phylogenetic tree based on the 16S rRNA gene grouped the 32 isolates into four groups (namely, Groups I, II, III and IV) within the genus *Bradyrhizobium* (Fig. 3). Isolate TUTGMeS6 from New Heavens, together with isolates TUTGMeS7 and TUTAHeS27 from the Malkerns Research Station grouped together (99.1–100% sequence similarity) and shared 99.1–100% sequence similarity with the type strains of *B. ganzhouense*, *B. campsiandrae*, *B. rifense* and *B*. *arachidis* (Fig. 3). Isolates TUTAHeS3, TUTAHeS29 and TUTAHeS26 from the Malkerns Research Station in Group II shared 99.1–100% sequence similarity among themselves and 98.9–100% sequence similarity with the reference type strains of *B. huanghuaihaiense, B. ingae, B. sacchari* and *B. betae* (Fig. 3). Furthermore, isolates TUTAHeS3 and TUTAHeS29 in Group II shared 100% sequence similarity with the reference type strains of *B. huanghuaihaiense*, *B. ingae*, *B*. *iriomotense* and *B*. *sacchari* with 54% bootstrap support. Isolates TUTAHeS4, TUTAHeS90 and TUTAHeS95 grouped with *B. canariense* and *B. lupine* in Group III with 99.3–100% sequence similarity. Cluster IV comprised 21 isolates from the Malkerns Research Station, one isolate (TUTGMeS4) from New Heaven and another isolate (TUTGMeS3) from Luve; and they shared 94.7 to 100% sequence similarity with *B. pachyrhizi*, *B. brasilense*, *B tropiciagri* and *B*. *elkanii* (Fig. 3).

### Phylogenetic relationship of isolates inferred from housekeeping (*atpD, dnaK, glnll, rpoB*) genes

Four housekeeping genes (*atpD, dnaK, glnII* and *rpoB*) were selected for a robust multilocus sequence phylogenetic analysis. The PCR amplification of the *atpD*, *dnaK*, *glnII* and *rpoB* genes yielded band sizes of 600 bp, 650 bp, 700 bp and 700 bp, respectively. However, final lengths of 419 bp, 214 bp, 433 bp and 314 bp were used to construct the phylogenies of *atpD*, *dnaK*, *glnII* and *rpoB*, respectively. With a few exceptions, the maximum likelihood phylogenetic trees from the individual housekeeping genes were consistent with the phylogram from the 16S rRNA gene (Fig. 3; Supplementary Fig. S1–S4). For example, the isolates in Group IV in the 16S rRNA phylogeny also grouped together in the single gene phylogenies of the housekeeping genes (*atpD, dnaK, glnII* and *rpoB*) and showed closeness with *B. elkanii* and *B. pachyrhizi*; however, isolate TUTCEeS14 grouped with *B. elkanii* as an outgroup of the other isolates in the *atpD* gene phylogeny (Fig. 3; Supplementary Fig. S1–S4).

### Phylogenetic analysis inferred from concatenated sequences of *atpD-glnII-rpoB* genes

**Figure 4:**
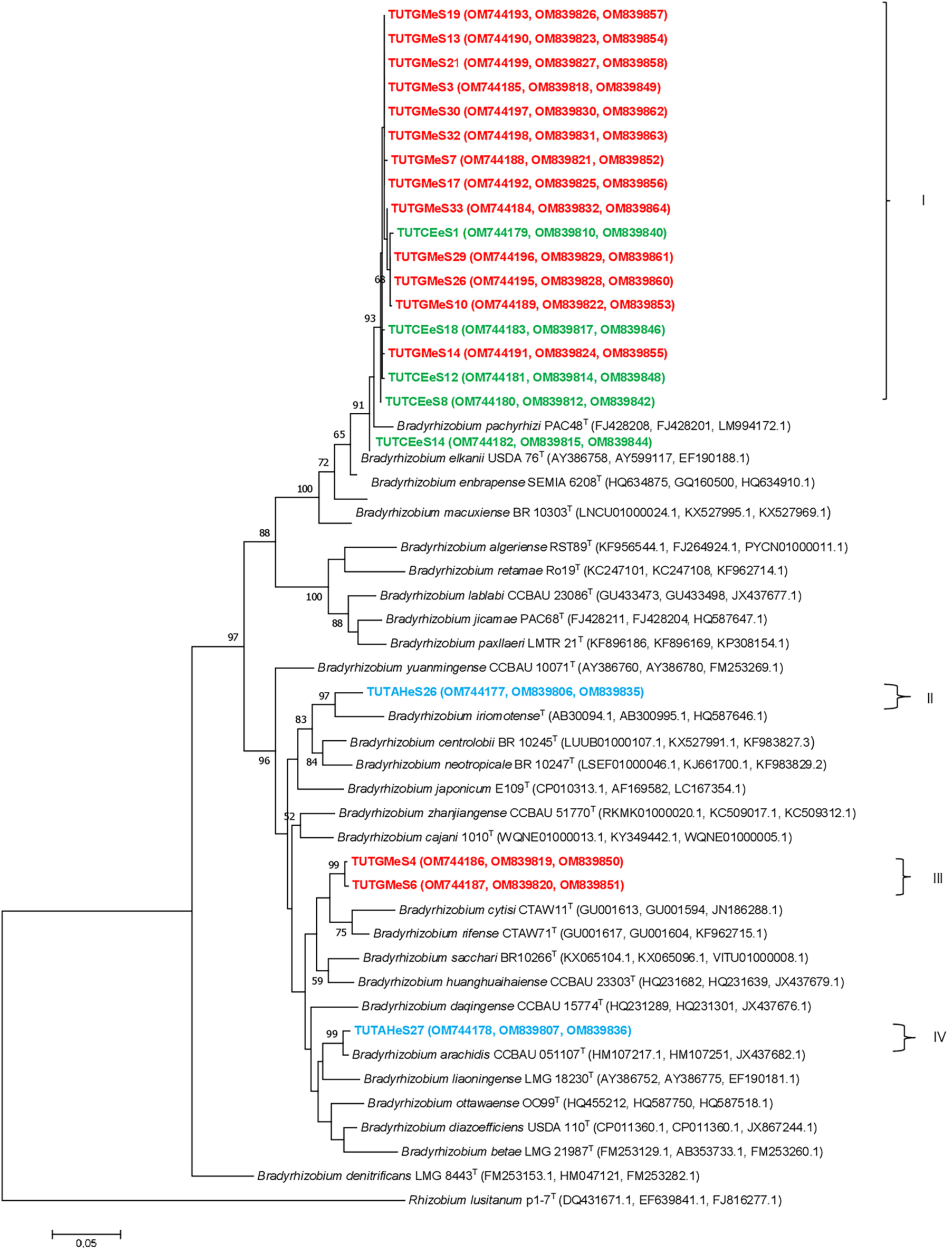
Maximum likelihood phylogenetic tree based on the concatenated *atpD-glnII-rpoB* gene sequences of groundnut, jack bean and soybean isolates from various locations in Eswatini. In each isolate the location is indicated by assigning different symbols, e.g., circle-Malkerns Research station; square-New Heaven and triangle-Luve. Isolates’ names are colour coded based on the host species as Blue-groundnut, Green-jack bean and Red-soybean.

Out of the 32 isolates that were selected for phylogenetic analysis, 22 of them yielded quality sequences for the *atpD, glnII* and *rpoB* genes, and were used to construct a concatenated tree based on those three genes (Fig. 4). The phylogram based on the concatenated gene sequences grouped the isolates into four main groups which were congruent with the phylogenies of the individual housekeeping genes (Fig. 4, Supplementary Fig. S1–S4). In Group I, thirteen soybean isolates and four jack been isolates grouped together with 89.5–100% sequence similarity (Fig. 4). Of the soybean isolates in this group, 12 originated from the Malkerns Research Station while one isolate was from Luve. All the four jack bean isolates in the group were from the Malkerns Research Station. Interestingly, all the isolates in Group I did not align with any reference type strains but shared 98.2–98.5% sequence similarity with *B. pachyrhizi*, the closest related type strain (Fig. 4).

Although isolate TUTCEeS14 grouped together with other isolates in Group I of the *glnII* and *rpoB* phylogenies, it stood separately together with the type strain *B. elkanii* as an outgroup of those isolates in Group I of the concatenated phylogenetic tree (Fig. 4; Supplementary Fig. S3, S4). In Group II of the concatenated tree, the groundnut isolate TUTAHeS26 grouped with the reference type strain *B. iriomotense* with 95.5% sequence similarity and 97% bootstrap support. Soybean isolates TUTGMeS4 and TUTGMeS6 from the New Heaven site in cluster III grouped together with 99.7% sequence similarity and 99% bootstrap support. The groundnut isolate TUTAHeS27 from Malkerns Research Station also grouped with the reference type strain of *B*. *arachidis* in Cluster IV, with 99.2% sequence similarity and 99% bootstrap support (Fig. 4).

### Phylogenetic analysis of isolates based on symbiotic (*nifH* and *nodC*) genes

**Figure 5:**
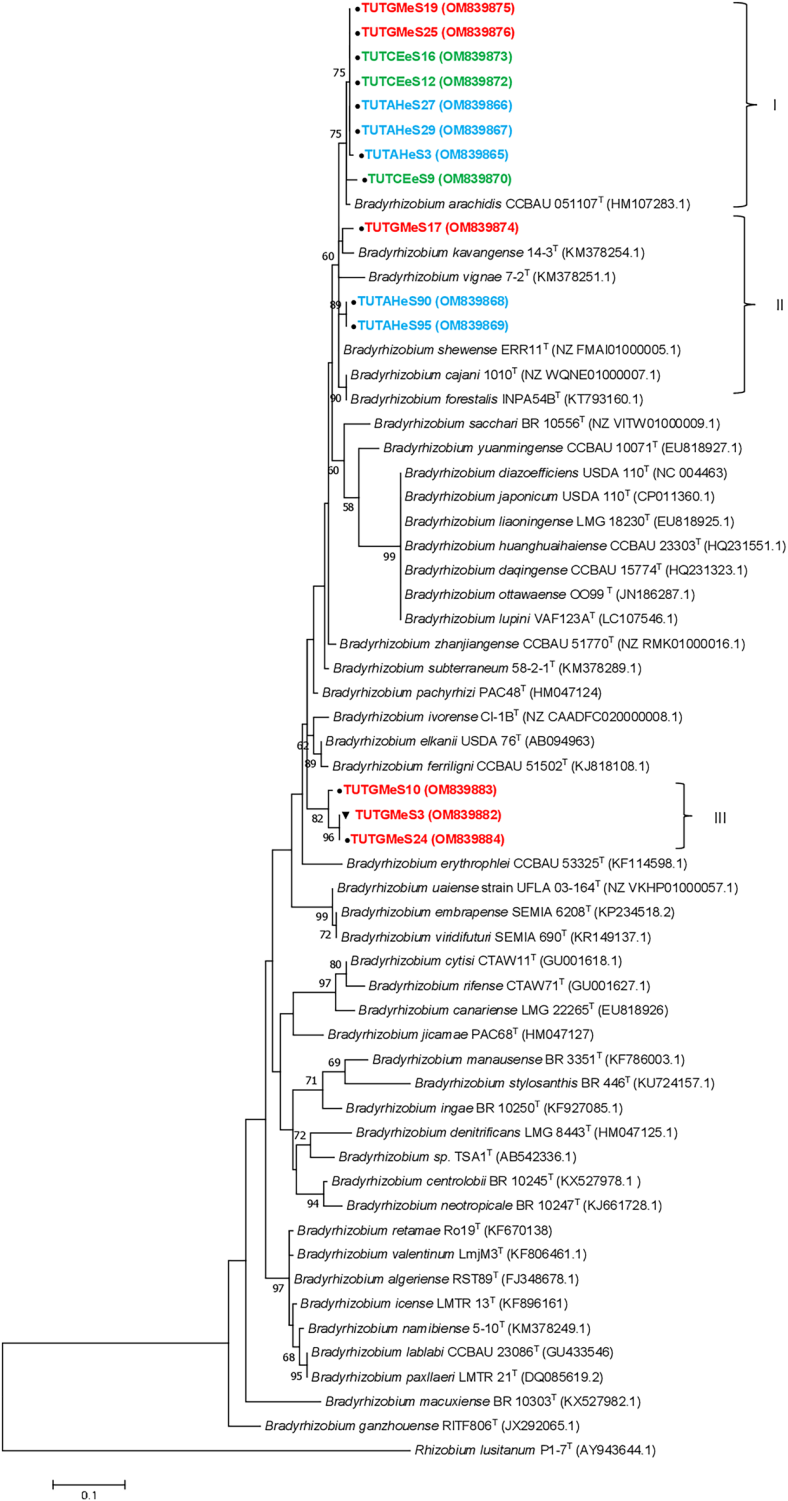
Maximum likelihood phylogenetic tree based on the *nifH* gene sequences of groundnut, jack bean and soybean isolates from various locations in Eswatini. For each isolate, the location of origin is indicated by assigning different symbols, e.g., circle-Malkerns Research station; square-New Heaven and triangle-Luve. GenBank accession numbers are indicated in parenthesis after the name of each isolate. Isolates’ names are colour coded based on the host species as Blue-groundnut, Green-jack bean and Red-soybean.

**Figure 6:**
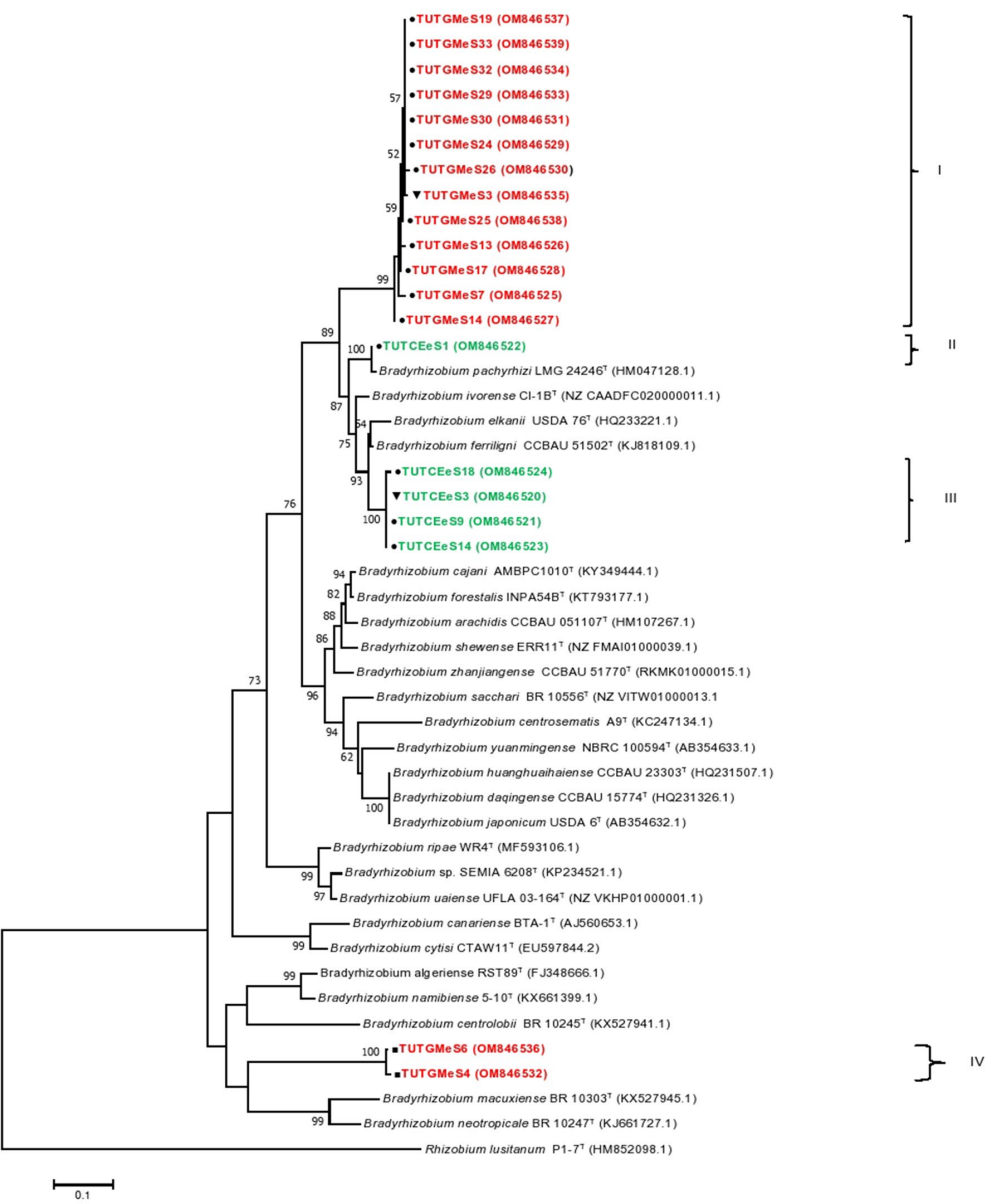
Maximum likelihood phylogenetic tree based on the *nodC* gene sequences of groundnut, jack bean and soybean isolates from various locations in Eswatini. For each isolate, the location of origin is indicated by assigning different symbols, e.g., circle-Malkerns Research station; square-New Heaven and triangle-Luve. GenBank accession numbers are indicated in parenthesis after the name of each isolate. Isolates’ names are colour coded based on the host species as Blue-groundnut, Green-jack bean and Red-soybean.

Phylogenetic analysis based on the two symbiotic (*nifH* and *nodC*) genes grouped the test isolates into various clusters within the *Bradyrhizobium* genus (Fig. 5, 6). With the *nifH* phylogeny, the isolates were grouped into three groups (Fig. 5), while in the *nodC* phylogeny, they formed four groups (Fig. 6). The phylogenies based on the *nifH* and *nodC* genes were distinct from each other and incongruent with the housekeeping gene phylogenies, though some isolates consistently grouped together in both the housekeeping and symbiotic gene phylogenies. For example, isolates TUTGMeS6 and TUTGMeS4 in Group IV of the *nodC* phylogeny were consistently grouped in the phylogenies based on housekeeping genes and symbiotic genes (Fig. 6; Supplementary Fig. S1, S4). In the *nifH* phylogeny, eight isolates from the Malkerns Research Station formed Group I and shared 98.0–99% sequence similarity with *B. arachidis* (Fig. 5). Moreover, the soybean isolate TUTGMeS17 and groundnut isolates TUTAHeS90 and TUTAHeS95 grouped together in Group II of the *nifH* phylogeny and shared 95.5–99.0% sequence similarity with the reference type strains *B. kavangense, B. vignae, B. shewense, B. cajani* and *B. forestalis* (Fig. 5). Furthermore, soybean isolates TUTGMeS10 and TUTGMeS24 from the Malkerns Research Station and isolate TUTGMeS3 from Luve formed Group III of the *nifH* phylogeny, with a 98.5 to 100% sequence similarity and 88% bootstrap support (Fig. 5).

In the *nodC* phylogeny, Group I contained 13 soybean isolates from the Malkerns Research Station that shared 87.0–100% sequence similarity, but stood away from any reference type strains, and shared only 87.0–88.2% sequence similarity with *B. pachyrhizi*, the closest related reference type strain (Fig. 6). Isolate TUTCEeS1 from the root nodules of jack bean grouped with the reference type strain of *B. pachyrhizi* in Group II of the *nodC* phylogeny with 99.1% sequence similarity and 100% bootstrap support. Group III consisted of four jack bean isolates from the Malkerns Research Station that shared 99.4–100% sequence similarity and 100% bootstrap support; these isolates shared 91.6–94.2% sequence similarity with *B. elkanii*, the closest reference type strain (Fig. 6). Soybean isolates TUTGMeS6 and TUTGMeS4 from the New Heaven site formed Group IV with 99.1% sequence similarity and 100% bootstrap support (Fig. 6).

## Discussion

To explore the genetic diversity and phylogenetic relationships among rhizobial symbionts of groundnut (*Arachis hypogea*), jack bean (*Canavalia ensiformis*) and soybean (*Glycine max*) in soils from Eswatini, ERIC PCR fingerprinting and multilocus sequence typing was carried out on authenticated rhizobial isolates of the test legume species. These isolates showed marked differences in colony growth, colour, shape, and size/diameter. The ERIC-PCR profiles of the isolates from the three legumes revealed a high genetic diversity as they constituted 56 ERIC-PCR types if considered at a 70% similarity level (Fig. 1; Table 1). Except for a recent report by Dlamini et al.^25^ which observed high genetic diversity among the symbionts of Bambara groundnut in Eswatini, no information could be retried on the diversity of rhizobia nodulating other legumes in this Southern African country. Nevertheless, the fact that Africa is a hotspot of rhizobial diversity is well documented for several grain legumes^5,10,12^. Since the genetic diversity of rhizobia can be influenced by the legume host and soil chemical properties of locations^26–29^, the general tendency for isolates to group together in the dendrogram constructed from their ERIC-PCR profiles based on legume host or geographic origin was expected (Fig. 1). Nevertheless, the fact that several Clusters contained rhizobial isolates from different legume species and locations suggests a certain commonality among the test legumes in terms of their preferred symbionts. A study by Chidebe et al.^1^ earlier found that the distribution of rhizobial isolates across clusters was not solely dependent on geographic origin or legume variety. Aside rhizobia, several non-rhizobial bacteria were isolated from the root nodules of the legumes tested, an observation similar to that observed by Mbah et al.^30^ in the root nodules of cowpea from South Africa. These non-rhizobial endophytes could be subject of future studies to assess their potential plant-growth promoting traits.

The distribution of rhizobia between geographic locations is often shaped by several soil physico-chemical properties^29^. For example, in this study, whereas the distribution of rhizobial symbionts at the Malkerns location was highly influenced by the levels of Ca, K, Mg and Fe in soils at the site, the microsymbionts at the New Heaven location were more influenced by P and K while those at Ka-Zulu were largely influenced by soil pH, Zn and Cu (Fig. 2). Whereas the observed influence of soil chemical parameters on the distribution of microsymbionts between different geographic locations has been reported for various grain legumes, these factors could also explain the close genetic similarities among microsymbionts from the same location^25–28^. It would therefore seem that the genetic fingerprints of the diverse rhizobia in African is linked to the equally diverse physico-chemical properties of soils that characterize various environments across the continent.

To assess the phylogenetic relationships among the diverse rhizobia isolated from the three test legumes in Eswatini, the 16S rRNA, housekeeping (*atpD, dnaK, glnII* and *rpoB*) and symbiotic (*nifH* and *nodC*) genes were sequenced and analysed. Analysis of the 16S rRNA gene sequences aligned all the isolates from the three legumes to species in the genus *Bradyrhizobium*. Though *Bradyrhizobium* species are well established symbionts of groundnut^4,31^ and soybean^32^ as found in this study, no previous reports could be retrieved regarding the rhizobial symbionts of jack bean. Thus, based on this study, *Bradyrhizobium* species are the preferred symbionts of this underutilized legume in African soils. The observed clustering of the groundnut isolates TUTAHeS3, TUTAHeS29 and TUTAHeS26 with *B. iriomotense* and *B. arachidis* was similarly reported by Chen et al.^33^ as well as Jaiswal et al.^4^. It was interesting to note that most of the jack bean and soybean isolates grouped with *B. elkanii* and *B. pachyrhizi* in this study, suggesting that the bradyrhizobial symbionts of these test legumes may share higher phylogenetic similarity when compared to their counterparts nodulating groundnut in the test locations.

The single gene phylograms of the test rhizobial isolates were congruent with each other, as well as with the phylogram based on the 16S rRNA gene. Consequently, the phylogeny based on concatenated sequences of *atpD-glnII-rpoB* refined the clustering of the isolates. For example, the soybean and jack bean isolates in Cluster I of the concatenated tree also grouped together in the 16S rRNA phylogram and in the phylogram based on the individual housekeeping genes (Figs. 3, 4; Supplementary Fig. S1–S4). Although the soybean and jack bean isolates in Cluster I of the concatenated tree did not group with any reference type strain, they shared 97.4–98.3% sequence similarity with *B. elkanii* and *B*. *pachyrhizi* (Fig. 4). These results are similar to those of Gyogluu et al.^12^ who also isolated *B. elkanii* and *B. pachyrhizi* from the root nodules of soybean in Mozambican soils.

Although the concatenated *atpD-glnII-rpoB* and individual housekeeping gene phylogenies were mostly congruent, those based on the symbiotic genes *nifH* and *nodC* genes were largely incongruent. For example, the jack bean and soybean isolates that formed Group I in the concatenated *atpD-glnII-rpoB* gene phylogeny were distributed in three different clusters in both the *nifH* and *nodC* phylogenies. Again, whereas these isolates did not group with reference type strains in the concatenated tree, they were closely aligned with the reference type strains of *B. pachyrhizi, B. elkanii* and *B. japonicum* in the *nifH* (93–95% sequence similarity) and *nodC* (79.3–99.1% sequence similarity) phylogenies. Furthermore, although the groundnut isolates grouped separately in the housekeeping gene and concatenated *atpD-glnII-rpoB* gene phylogenies, they clustered together with the jack bean and soybean isolates in the *nifH* phylogeny. Incongruencies among single gene phylogenies as observed in this study are largely attributed to the frequency of horizontal gene transfer among prokaryotes^34^. Among rhizobia for example, the acquisition of symbiotic genes from distantly related bacteria as well as differences in evolutionary processes of chromosomes can partly contribute to the observed inconsistencies in the phylogenies of single genes^35^. Similar discrepancies in phylogenies of housekeeping and symbiotic genes were also observed in earlier reports on the symbionts of legumes such as cowpea and Soybean^1,12^.

## Conclusion

The findings of this study revealed a high genetic diversity among the bradyrhizobial symbionts of groundnut, jack bean and soybean in the soils of Eswatini. A few of the rhizobial symbionts of the test legumes belonged to specie in the *Bradyrhizobium* genus, while others showed high divergence from the known reference type strains based on *16S rRNA*, *atpD*, *glnII*, *rpoB* and *dnaK* gene sequences. Moreover, based on the symbiotic gene sequences, a few symbionts of the test legumes showed close alignment with *B. arachidis* and *B. kavangense* in the *nifH* phylogeny, while only the jack bean isolate TUTCEeS1 aligned with *B. pachyrhizi* in the *nodC* phylogram. Thus, most of the rhizobial symbionts evaluated were highly divergent from the known symbiovars. In the absence of any retrievable information on the rhizobia nodulating jack bean, these findings could be the first report of the crop’s microsymbionts in an African soil.

## Supplementary Information

Supplementary Information.

## Data Availability

The nucleotide sequences of all the tested genes were submitted to the NCBI GenBank database to obtain the accession numbers: OM721967–OM721998 (16S rRNA), OM744177–OM744199 (*atpD*), OM839789–OM839804 (*dnaK*), OM839805–OM839832 (*glnII*), OM839833–OM839864 (*rpoB*), OM839865–OM839889 (*nifH*) and OM846520–OM846539 (*nodC*).
