# Supplementary material for: Phylogenetic relationships among Bradyrhizobium species nodulating groundnut (Arachis hypogea L.), jack bean (Canavalia ensiformis L.) and soybean (Glycine max Merr.) in Eswatini
Source: Sci Rep. 2022 Jun 23;12:10629. doi: 10.1038/s41598-022-14455-9 (PMC9226157; doi:10.1038/s41598-022-14455-9)
Supplement: Supplementary file 1 — Supplementary Information. [file 41598_2022_14455_MOESM1_ESM.docx]

Phylogenetic relationships among *Bradyrhizobium* Species Nodulating Groundnut (*Arachis hypogea* L.), jack bean (*Canavalia ensiformis* L.) and soybean (*Glycine max* Merr.) in Eswatini

Zanele D Ngwenya^1^, Mustapha Mohammed^2,3^, Sanjay K Jaiswal^2^, Felix D Dakora^2^

^1^Department of Crop Sciences and ^2^Department of Chemistry, Tshwane University of Technology, Private Bag X680, Pretoria, 001, South Africa

^3^Department of Crop Science, University for Development Studies, P.O. Box 1350, Tamale, Ghana

*Corresponding author:

Felix D. Dakora

Chemistry Department, Tshwane University of Technology, Private Bag X680, Pretoria 0001, South Africa.

Tel: +27 12 382 6120

Fax: +27 12 382 6286

Email: [DakoraFD@tut.ac.za](mailto:DakoraFD@tut.ac.za)

Supplementary Table S1. Chemical properties of bulk soils from Ka-Zulu, Luve and New Heaven farmers’ fields, and from the Malkerns Research Station in the 2016/2017 cropping season

| **Nutrient** | **Ka-Zulu Farm** | **New Heaven** | **Luve** | **Malkerns** |
| --- | --- | --- | --- | --- |
| pH (KCl) | 4.3 | 3.9 | 6.1 | 4.4 |
| Total N (%) | 0.069 | 0.112 | 0.036 | 0.075 |
| Available P (mg kg^-1^) | 4 | 13 | 53 | 14 |
| K (mg kg^-1^) | 25 | 41 | 21 | 54 |
| Cu (mg kg^-1^) | 1.47 | 5.9 | 0.14 | 0.91 |
| Zn (mg kg^-1^) | 2.17 | 1.68 | 2.09 | 1.69 |
| Ca (mg kg^-1^) | 0.22 | 1.09 | 0.35 | 1.15 |
| Fe (mg kg^-1^) | 54.69 | 152.1 | 81.66 | 452.80 |
| Mg cmol(+) kg^-1^ | 0.13 | 0.81 | 0.18 | 0.84 |
| Na (mg kg^-1^) | 7 | 25 | 9 | 5 |

Supplementary Table S2 Primers and temperature profiles used in DNA amplification of genes

| **Target gene** | **Primer sequences 5' - 3'** | **Temperature profiles** | | **References** | |
| --- | --- | --- | --- | --- | --- |
| ERIC | ATGTAAGCTCCTGGGGATTCAC | | 5 min 95 °C, 30 × (30 s 94 | | ^1^ |
|  | AAGTAAGTGACTGGGGTGAGCG | | °C, 1 min 52 °C, 8 min 65 | |  |
|  |  | | ◦C), 16 min 65 °C | |  |
|  |  | |  | |  |
| 16S rRNA | AGAGTTTGATCCTGGCTCAG | | 2 min 95 °C, 30 X (15 s 94 | | ^2^ |
|  | CTTAAGGAGGTGATCCAGCC | | °C, 45 s 93°C, 45 s 55°C, 2 | |  |
|  |  | | min 72 °C) 5 min 72 °C. | |  |
|  |  | |  | |  |
| *atpD* | TCTGGTCCGYGGCCAGGAAG | | 2 min at 95°C, 35 x (45s at | | ^3^ |
|  | CGACACTTCCGARCCSGCCTG | | 95°C, 30s at 65°C, 1.5min at | |  |
|  |  | | at 72°C), 10 mins at 72°C | |  |
|  |  | |  | |  |
| *dnaK* | GTACATGGCCTCGCCGAGCTTCA | | 1 min 94°C, 35 x (1 min 94°C, | | ^3^ |
|  | AAGGAGCAGCAGATCCGCATCCA | | 1 min 62°C, 40 s 72°C) | |  |
|  |  | |  | |  |
| *glnll* | AAGCTCGAGTACATCTGGCTCGACGG | | 2 min at 95°C, 35 X (45s at | | ^3^ |
|  | SGAGCCGTTCCAGTCGGTGGTGTCG | | 95°C, 30s at 65°C, 90s at | |  |
|  |  | | 72°C), 10 min at 72°C | |  |
|  |  | |  | |  |
| *rpoB* | ACATCGAGTTCGACGCCAAGG | | 5 min at 95°C; 20 x 45s at 95°C, | | ^4^ |
|  | CATTGACGTGGTCGATGTCG | | 30s at 60°C (-0.5°C per cycle) | |  |
|  |  | | and 1 min 30s at 72°C; 25 x 30s at | |  |
|  |  | | 25 x 30s at 94°C, 30s at 55°C, 1 min | |  |
|  |  | | 30s at 72°C; 10 min at 72°C | |  |
|  |  | |  | |  |
| *nifH* | TACGGNAARGGSGGNATCGGCAA | | 5 min 94 °C, 20 X [30 s 94 | | ^4^ |
|  | AGCATGTCYTCSAGYTCNTCCA | | °C, 30 s 65 °C (-0.5 °C / | |  |
|  |  | | cycle), 90 s 72 °C], 25 X (30 | |  |
|  |  | | s 94 °C, 30 s 65 °C, 90 s 72 | |  |
|  |  | | °C), 10 min 72 °C | |  |
|  |  | |  | |  |
| *nodC* | GTCGATTGCMRGTCAAGACTACG | | 30 s 94 °C, 40 × (30s 94 °C, | | ^5^ |
|  | GCCAGGTCTIGTTGCGATTGCTC | | 1 min 55.4 °C, 30s 72 °C), 5 | |  |
|  |  | | min 72 °C | |  |

■**TUTGMeS4 (OM744186)**

■**TUTGMeS6 (OM744187)**

*Bradyrhizobium daqingense* CCBAU 15774^T^ (HQ231289)

*Bradyrhizobium sacchari* BR10266^T^ (KX065104.1)

*Bradyrhizobium huanghuaihaiense* CCBAU 23303^T^ (HQ231682)

*Bradyrhizobium rifense* CTAW71^T^ (GU001617)

*Bradyrhizobium guangxiense* CCBAU 53363^T^ (KC508926)

*Bradyrhizobium centrosematis* A9 (KC247129.1)

*Bradyrhizobium liaoningense* LMG 18230^T^ (AY386752)

●**TUTAHeS27 (OM744178)**

*Bradyrhizobium arachidis* CCBAU 051107^T^ (HM107217.1)

*Bradyrhizobium betae* LMG 21987^T^ (FM253129.1)

*Bradyrhizobium shewense* ERR11^T^ (NZ FMAI01000019.1)

*Bradyrhizobium ottawaense* OO99^T^ (HQ455212)

*Bradyrhizobium diazoefficiens* SEMIA 5080^T^ (FJ390957.1)

*Bradyrhizobium denitrificans* LMG 8443^T^ (FM253153.1)

*Bradyrhizobium guangdongense* CCBAU 51649^T^ (KC508916)

*Bradyrhizobium ganzhouense* RITF807^T^ (JX277183)

*Bradyrhizobium cytisi* CTAW11^T^ (GU001613)

*Bradyrhizobium canariense* LMG 22265^T^ (FM253135)

*Bradyrhizobium cajani* 1010^T^ (NZ WQNE01000013.1)

*Bradyrhizobium centrolobii* BR 10245^T^ (NZ LUUB01000107.1)

*Bradyrhizobium zhanjiangense* CCBAU 51770^T^ (RKMK01000020.1)

*Bradyrhizobium oligotrophica* LMG 10732^T^ (JQ619232)

*Bradyrhizobium valentinum* LmjM3^T^ (JX518561)

*Bradyrhizobium algeriense* RST89^T^ (KF956544.1)

*Bradyrhizobium lablabi* CCBAU 23086^T^ (GU433473)

*Bradyrhizobium jicamae* PAC68^T^ (FJ428211)

*Bradyrhizobium paxllaeri* LMTR 21^T^ (KF896186)

*Bradyrhizobium namibiense* 5-10^T^ (KX661387.1)

*Bradyrhizobium retamae* Ro19^T^ (KC247101)

*Bradyrhizobium icense* LMTR 13^T^ (KF896192)

●**TUTAHeS26 (OM744177)**

*Bradyrhizobium japonicum bv.genistearum* BLup-MR1^T^ (AY386751.1)

*Bradyrhizobium forestalis* INPA54B^T^ (KF452722.1)

*Bradyrhizobium neotropicale* BR 10247^T^ (NZ LSEF01000046.1)

*Bradyrhizobium iriomotense*^T^ (AB300994.1)

*Bradyrhizobium yuanmingense* CCBAU 10071^T^ (AY386760)

*Bradyrhizobium tropiciagri* SEMIA 6148^T^ (FJ390968)

*Bradyrhizobium macuxiense* BR 10303^T^ (NZ LNCU01000024.1)

*Bradyrhizobium enbrapense* SEMIA 6208^T^ (HQ634875)

*Bradyrhizobium pachyrhizi* PAC48^T^ (FJ428208)

*Bradyrhizobium uaiense* UFLA03-164^T^ (KF452739.1)

●**TUTCEeS14 (OM744182)**

*Bradyrhizobium elkanii* USDA 76^T^ (AY386758)

●**TUTGMeS26 (OM744195)**

●**TUTGMeS29 (OM744196)**

●**TUTGMeS10 (OM744189)**

●**TUTCEeS1 (OM744179)**

●**TUTGMeS33 (OM744184)**

●**TUTCEeS8 (OM744180)**

*Bradyrhizobium brasilense* UFLA03-290^T^ (KF452733.1)

●**TUTCEeS12 (OM744181)**

●**TUTCEeS18 (OM744183)**

▼**TUTGMeS3 (OM744185)**

**●TUTGMeS7 (OM744188)**

**●TUTGMeS13 (OM744190)**

●**TUTGMeS14 (OM744191)**

**●TUTGMeS17 (OM744192)**

●**TUTGMeS19 (OM744193)**

●**TUTGMeS25 (OM744194)**

●**TUTGMeS30 (OM744197)**

**●TUTGMeS32 (OM744198)**

**●TUTGMeS21 (OM744199)**

*Rhizobium lusitanum* p1-7^T^ (DQ431671.1)

99

97

93

97

74

54

92

50

81

57

86

66

72

0.1

I

II

III

Supplementary Fig. S1. Maximum likelihood phylogenetic tree of groundnut, jack bean and soybean isolates from various locations in Eswatini based on *atpD* gene sequences. For each isolate, the location of origin is indicated by assigning different symbols, e.g., circle-Malkerns Research station; square-New Heaven and triangle-Luve. GenBank accession numbers are indicated in parenthesis after the name of each isolate. Isolates are colour coded based on the host species as Blue-groundnut, Green-jack bean and Red-soybean.

**■TUTGMeS4 (OM839795)**

■**TUTGMeS6 (OM839796)**

*Bradyrhizobium cytisi* CTAW11^T^ (KF532219.1)

*Bradyrhizobium rifense* CTAW71^T^ (JQ945187.1)

*Bradyrhizobium iriomotense* LMG 24129^T^ (JF308944.1)

*Bradyrhizobium cajani* 1010^T^ (NZ WQNE01000019.1)

*Bradyrhizobium manausense*  BR 3351^T^ (KF786001.1)

*Bradyrhizobium ingae* BR 10250^T^ (KF927055.1)

*Bradyrhizobium neotropicale* BR 10247^T^ (KJ661693.1)

*Bradyrhizobium centrolobii* BR 10245^T^ (KX527928.1)

*Bradyrhizobium subterraneum* 2-1^T^ (KP308157.1)

*Bradyrhizobium vignae* 7-2^T^ (KR259951.1)

*Bradyrhizobium canariense* BTA-1^T^ (AY923047.1)

*Bradyrhizobium diazoefficiens* SEMIA 5060^T^ (JX867240.1)

*Bradyrhizobium kavangense* 14-3^T^ (KR259949.1)

*Bradyrhizobium liaoningense*  LMG 18230^T^ (AY923041.1)

*Bradyrhizobium stylosanthis* BR 446^T^ (KU724145.1)

*Bradyrhizobium daqingense* CCBAU 15774^T^ (KF962684.1)

*Bradyrhizobium arachidis* CCBAU 051107^T^ (JX437668.1)

*Bradyrhizobium yuanmingense* CCBAU 10071^T^ (AY923039.1)

*Bradyrhizobium huanghuaihaiense* CCBAU 23303^T^ (KF962686.1)

*Bradyrhizobium sacchari* BR10266^T^ (KX065100.1)

*Bradyrhizobium lupini* DnaK^T^ (MN525206.1)

*Bradyrhizobium denitrificans* LMG 8443^T^ (KF962685.1)

*Bradyrhizobium oligotrophicum* S5 LMG 10732^T^ (KF962688.1)

*Bradyrhizobium namibiense* 5-10^T^ (KP402058.1)

*Bradyrhizobium ivorense* CI-1B^T^ (MK376329.1)

*Bradyrhizobium macuxiense* BR 10303^T^ (KX527932.1)

*Bradyrhizobium ripae* WR4^T^ (MF593102.1)

*Bradyrhizobium uaiense* UFLA03-164^T^ (KF452780.1)

*Bradyrhizobium tropiciagri* SEMIA 6148^T^ (FJ391008.1)

*Bradyrhizobium viridifuturi* SEMIA 690^T^ (KR149128.1)

*Bradyrhizobium mercantei* SEMIA 6399^T^ (KX690617.1)

●**TUTCEeS1 (OM839789)**

●**TUTCEeS8 (OM839791)**

●**TUTGMeS13 (OM839797)**

●**TUTGMeS14 (OM839798)**

●**TUTGMeS19 (OM839799)**

●**TUTGMeS25 (OM839800)**

●**TUTGMeS30 (OM839801)**

▼**TUTGMeS3 (OM839804)**

●**TUTCEeS9 (OM839792)**

●**TUTCEeS18 (OM839794)**

●**TUTGMeS26 (OM839802)**

●**TUTGMeS29 (OM839803)**

●**TUTCEeS3 (OM839790)**

*Bradyrhizobium forestalis* INPA54B^T^ (KF452796.1)

**●TUTCEeS14 (OM839793)**

*Bradyrhizobium pachyrhizi* PAC48^T^ (JN207406.1)

*Bradyrhizobium elkanii* USDA 76^T^ (AY328392.1)

*Bradyrhizobium algeriense* RST89^T^ (FJ264922.1)

*Bradyrhizobium icense* LMTR 13^T^ (KF896182.1 )

*Bradyrhizobium lablabi* CCBAU 23086^T^ (KF896185.1)

*Bradyrhizobium paxllaeri* LMTR 21^T^ (AY923038.1)

*Bradyrhizobium retamae* Ro19^T^ (KF896184.1)

*Rhizobium tropici* strain H12^T^ (EU488760.1)

97

70

89

73

63

57

66

62

64

58

84

62

0.1

I

II

Supplementary Fig. S2. Maximum likelihood phylogenetic tree of groundnut, jack bean and soybean isolates from various locations in Eswatini based on *dnaK* gene sequences. For each isolate the location is indicated by assigning different symbols, e.g., circle-Malkerns Research station; square-New Heaven and triangle-Luve. GenBank accession numbers are indicated in parenthesis after the name of each isolate. Isolates’ names are colour coded based on the host species as Blue-groundnut, Green-jack bean and Red-soybean.

*Bradyrhizobium cytisi* CTAW11^T^ (GU001594)

*Bradyrhizobium rifense* CTAW71^T^ (GU001604)

*Bradyrhizobium ganzhouense* RITF807^T^ (JX277111)

**■TUTGMeS4 (OM839819)**

■**TUTGMeS6 (OM839820)**

*Bradyrhizobium ingae* BR 10250^T^ (KF927067)

*Bradyrhizobium lupini* USDA 3051^T^ (KM114862)

*Bradyrhizobium iriomotense*^T^  (AB300995.1)

●**TUAHeS95 (OM839809)**

*Bradyrhizobium japonicum*^T^ (AF169582)

*Bradyrhizobium cajani* AMBPC1010^T^ (KY349442.1)

*Bradyrhizobium betae* LMG 21987^T^ (AB353733.1)

*Bradyrhizobium diazoefficiens* USDA 110^T^ (CP011360.1)

*Bradyrhizobium ottawaense* OO99^T^ (HQ587750)

*Bradyrhizobium daqingense* CCBAU 15774^T^ (HQ231301)

*Bradyrhizobium zhanjiangense* CCBAU 51778^T^ (KC509017.1)

*Bradyrhizobium yuanmingense* CCBAU 10071^T^ (AY386780)

*Bradyrhizobium centrolobii* BR 10245^T^ (KX527991.1)

*Bradyrhizobium neotropicale* BR 10247^T^ (KJ661700.1)

●**TUTAHeS26 (OM839806)**

*Bradyrhizobium arachidis* CCBAU 051107^T^ (HM107251)

●**TUTAHeS27 (OM839807)**

●**TUTAHeS3 (OM839805)**

●**TUTAHeS29 (OM839808)**

*Bradyrhizobium huanghuaihaiense* CCBAU 23303^T^ (HQ231639)

*Bradyrhizobium sacchari* BR10266^T^ (KX065096.1)

*Bradyrhizobium guangdongense* CCBAU 51649^T^ (KC509023)

*Bradyrhizobium manausense* BR 3351^T^ (KF785986)

*Bradyrhizobium vignae* 7-2^T^ (KM378443)

*Bradyrhizobium kavangense* 14-3^T^ (KM378446)

*Bradyrhizobium liaoningense bv. glycinearum* LMG 18230^T^ (AY386775)

*Bradyrhizobium guangxiense* CCBAU 53363^T^ (KC509033)

*Bradyrhizobium centrosematis* A9^T^ (KX012940.1)

*Bradyrhizobium namibiense* 5-10^T^ (KM378440.1)

*Bradyrhizobium oligotrophica* LMG 10732^T^ (JQ619233)

*Bradyrhizobium denitrificans* LMG 8443^T^ (HM047121)

*Bradyrhizobium valentinum* LmjM3^T^ (JX518575)

*Bradyrhizobium algeriense* RST89^T^ (FJ264924.1)

*Bradyrhizobium jicamae* PAC68^T^ (FJ428204)

*Bradyrhizobium lablabi* CCBAU 23086^T^ (GU433498)

*Bradyrhizobium paxllaeri* LMTR 21^T^ (KF896169)

Bradyrhizobium retamae Ro19 (KC247108)

*Bradyrhizobium icense* LMTR 13 (KF896175.1)

*Bradyrhizobium erythrophlei* CCBAU 53325^T^ (KF114693)

*Bradyrhizobium macuxiense* BR 10303^T^ (KX527995.1)

*Bradyrhizobium elkanii* USDA 76^T^ (AY599117)

*Bradyrhizobium viridifuturi* SEMIA 690^T^ (KR149131)

*Bradyrhizobium enbrapense* SEMIA 6208^T^ (GQ160500)

*Bradyrhizobium tropiciagri* SEMIA 6148^T^ (FJ391048)

*Bradyrhizobium ferriligni* CCBAU 51502^T^ (KJ818099)

*Bradyrhizobium pachyrhizi* PAC48^T^ (FJ428201)

●**TUTCEeS9 (OM839813)**

**●TUTGMeS7 (OM839821)**

●**TUTCEeS12 (OM839814)**

●**TUTGMeS32 (OM839831)**

●**TUTCEeS1 (OM839810)**

●**TUTCEeS3 (OM839811)**

●**TUTCEeS8 (OM839812)**

●**TUTCEeS14 (OM839815)**

●**TUTCEeS16 (OM839816)**

●**TUTCEeS18 (OM839817)**

▼**TUTGMeS3 (OM839818)**

●**TUTGMeS10 (OM839822)**

**●TUTGMeS13 (OM839823)**

●**TUTGMeS14 (OM839824)**

●**TUTGMeS17 (OM839825)**

●**TUTGMeS19 (OM839826)**

●**TUTGMeS26 (OM839828)**

●**TUTGMeS29 (OM839829)**

●**TUTGMeS21 (OM839827)**

●**TUTGMeS30 (OM839830)**

●**TUTGMeS33 (OM839832)**

*Rhizobium lusitanum* P1-7^T^ (EF639841.1)

99

84

53

95

84

51

90

69

60

69

88

70

92

60

51

94

63

92

0.1

I

II

III

IV

Supplementary Fig. S3 Maximum likelihood phylogenetic tree of groundnut, jack bean and soybean isolates from various locations in Eswatini based on *glnII* gene sequences. For each isolate, the location of origin is indicated by assigning different symbols, e.g., circle-Malkerns Research station; square-New Heaven and triangle-Luve. GenBank accession numbers are indicated in parenthesis after the name of each isolate. Isolates’ names are colour coded based on the host species as Blue-groundnut, Green-jack bean and Red-soybean.

●**TUTGMeS33 (OM839864)**

●**TUTGMeS7 OM839852)**

●**TUTGMeS32 (OM839863)**

●**TUTGMeS30 (OM839862)**

▼**TUTGMeS3 (OM839849)**

●**TUTGMeS29 (OM839861)**

●**TUTGMeS26 (OM839860)**

●**TUTGMeS25 (OM839859)**

●**TUTGMeS21 (OM839858)**

●**TUTGMeS19 (OM839857)**

●**TUTGMeS10 (OM839853)**

●**TUTGMeS13 (OM839854)**

●**TUTGMeS17 (OM839856)**

●**TUTGMeS14 (OM839855)**

●**TUTCEeS16 (OM839845)**

●**TUTCEeS18 (OM839846)**

*Bradyrhizobium braselense* UFLA03-321^T^ (KF452879.1)

*Bradyrhizobium pachyrhizi* PAC48T^T^ (LM994172.1)

●**TUTCEeS1 (OM839840)**

●**TUTCEeS12 (OM839848)**

●**TUTCEeS14 (OM839844)**

●**TUTCEeS3 (OM839841)**

●**TUTCEeS8 (OM839842)**

●**TUTCEeS9 (OM839843)**

*Bradyrhizobium elkanii* USDA 76^T^ (EF190188.1)

●**TUTCEeS21 (OM839847)**

*Bradyrhizobium ripae* WR4^T^ (MF593098.1)

*Bradyrhizobium viridifuturi* SEMIA 690^T^ (KU724169.1)

*Bradyrhizobium embrapense* SEMIA 6208^T^ (HQ634910.1)

*Bradyrhizobium uaiense* UFLA 03-164^T^ (VKHP01000007.1)

*Bradyrhizobium ivorense* CI-1B^T^ (MK376336.1)

*Bradyrhizobium macuxiense* BR 10303^T^ (KX527969.1)

*Bradyrhizobium algeriense* RST91^T^ PYCN01000011.1)

*Bradyrhizobium retamae* Ro19^T^ (KF962714.1)

*Bradyrhizobium lablabi* CCBAU 23086^T^ (JX437677.1)

*Bradyrhizobium jicamae* LMG 24556^T^ (HQ587647.1)

*Bradyrhizobium paxllaeri* LMTR 21^T^ (KP308154.1)

*Bradyrhizobium denitrificans* LMG 8443T^T^ (FM253282.1)

*Bradyrhizobium oligotrophicum* S58 LMG 10732^T^ (KF962713.1)

*Bradyrhizobium ingae* BR 10250^T^ (KF927073.1)

●**TUTAH26 (OM839835)**

*Bradyrhizobium iriomotense* LMG 24129^T^ (HQ587646.1)

*Bradyrhizobium centrolobii* BR 10245^T^ (KF983827.3)

*Bradyrhizobium neotropicale* BR 10247^T^ (KF983829.2)

*Bradyrhizobium arachidis* CCBAU 051107^T^ (JX437682.1)

●**TUTAHeS3 (OM839833)**

●**TUTAHeS27 (OM839836)**

●T**UTAHeS29 (OM839837)**

*Bradyrhizobium ottawaense* OO99^T^ (HQ587518.1)

*Bradyrhizobium shewense* ERR11^T^ (FMAI01000007.1)

*Bradyrhizobium zhanjiangense* CCBAU 51778^T^ (KC509312.1)

*Bradyrhizobium cajani* 1010^T^ (WQNE01000005.1)

*Bradyrhizobium sacchari* BR 10555^T^ (VITU01000008.1)

*Bradyrhizobium daqingense* CCBAU 15774^T^ (JX437676.1)

*Bradyrhizobium liaoningense* LMG 18230T^T^ (EF190181.1)

*Bradyrhizobium subterraneum* 54 1-1^T^ (KM378344.1)

*Bradyrhizobium forestalis* INPA54B^T^ (PGVG01000026.1)

*Bradyrhizobium yuanmingense* LMG 21827T^T^ (FM253269.1)

*Bradyrhizobium vignae* 7-2^T^ (KM378308.1)

*Bradyrhizobium kavangense* 14-3^T^ (KM378311.1)

*Bradyrhizobium huanghuaihaiense* CCBAU 23303^T^ (JX437679.1)

*Bradyrhizobium manausense* BR 3351^T^ (KF785998.1)

*Bradyrhizobium stylosanthis* BR 446^T^ (KU724166.1)

*Bradyrhizobium diazoefficiens* SEMIA 6059^T^ (JX867244.1)

■**TUTGMeS4 (OM839850)**

■**TUTGMeS6 (OM839851)**

*Bradyrhizobium cytisi* LMG 25866^T^ (JN186288.1)

*Bradyrhizobium rifense* CTAW71^T^ (KF962715.1)

*Bradyrhizobium betae* LMG 21987T^T^ (FM253260.1)

*Bradyrhizobium canariense* LMG 22265T^T^ (FM253263.1)

**●TUTAHeS4 (OM839834)**

*Bradyrhizobium japonicum* USDA 6^T^ (LC167354.1)

●**TUTAHeS90 (OM839838)**

●**TUTAHeS95 (OM839839)**

*Rhizobium lusitanum* P1-7^T^ (FJ816277.1)

82

90

96

57

98

88

87

90

75

62

76

53

65

68

85

80

70

94

71

55

77

86

70

78

70

69

63

0,05

II

I

III

IV

V

Supplementary Fig. S4 Maximum likelihood phylogenetic tree of groundnut, jack bean and soybean from various locations in Eswatini based on *rpoB* gene sequences. For each isolate, the location of origin is indicated by assigning different symbols, e.g., circle-Malkerns Research station; square-New Heaven and triangle-Luve. GenBank accession numbers are indicated in parenthesis after the name of each isolate. Isolates’ names are colour coded based on the host species as Blue-groundnut, Green-jack bean and Red-soybean.

**References**

1. Versalovic, J., Schneider, M., De Bruihn, F. J. & Lupski, J. R. Genomic fingerprinting of bacteria using Repetitive Sequence-Based ploymerase chain reaction. *J. Clin. Microbiol.* **5**, 25–40 (1994).

2. Weisburg, W. G., Barns, S. M., Pelletier, D. A. & Lane, D. J. 16S ribosomal DNA amplification for phylogenetic study. *J. Bacteriol.* **173**, 697–703 (1991).

3. Stepkowski, T. *et al.* *Bradyrhizobium canariense* and *Bradyrhizobium japonicum* are the two dominant rhizobium species in root nodules of lupin and serradella plants growing in Europe. *Syst. Appl. Microbiol.* **34**, 368–375 (2011).

4. Nzoué, A. *et al.* Multilocus sequence analysis of bradyrhizobia isolated from Aeschynomene species in Senegal. *Syst. Appl. Microbiol.* **32**, 400–412 (2009).

5. Laguerre, G. *et al.* Classification of rhizobia based on nodC and nifH gene analysis reveals a close phylogenetic relationship among *Phaseolus vulgaris* symbionts. *Microbiology* **147**, 981–993 (2001).
